# Soliton solution, breather solution and rational wave solution for a generalized nonlinear Schrödinger equation with Darboux transformation

**DOI:** 10.1038/s41598-023-36295-x

**Published:** 2023-06-09

**Authors:** Chengcheng Fan, Li Li, Fajun Yu

**Affiliations:** School of Mathematics and Systematic Sciences, Shenyang Normal University, Shenyang, 110034 China

**Keywords:** Mathematics and computing, Physics

## Abstract

In this paper, the exact solutions of generalized nonlinear Schrödinger (GNLS) equation are obtained by using Darboux transformation(DT). We derive some expressions of the 1-solitons, 2-solitons and *n*-soliton solutions of the GNLS equation via constructing special Lax pairs. And we choose different seed solutions and solve the GNLS equation to obtain the soliton solutions, breather solutions and rational wave solutions. Based on these obtained solutions, we consider the elastic interactions and dynamics between two solitons.

## Introduction

The generalized nonlinear Schrödinger(GNLS) equation is an important nonlinear evolution equation, which can describe physical models and phenomena, such as: the Bose–Einstein condensation, nonlinear optics, plasma physics condensed matter physics, fluid mechanics, and so on. Latchio Tiofack, Mohamadou and Kofan$\acute{e}$ considered the nonuniform $1+1$ dimensional coupled nonlinear schrödinger equations^1^, and presented some exact solutions by using the transformation. Vijayalekshmi, Mahalingam and Mani–Rajan studied the propagation of optical solitons in the nonautonomous nonlinear Schrödinger equation with a generalized external potential^2^. The nonlinear Schrödinger equation has been extended to various soliton models^3^ including variable coefficient, complex coefficient, high dimensional, high order, nonlocal and fractional order equations^4–6^. Some solitary wave solutions^7^, rogue wave solutions^8^, bright and dark solitons^9^ are derived in nonlinear Schrödinger equation.

There are many methods to solve soliton equation, such as Hirota bilinear method^10,11^, inverse scattering method^12,13^, homogeneous balance method^14,15^, Darboux transform (DT) method^16,17^ and so on. Some solutions are successfully solved in different types of partial differential equations via these above methods. Some higher-order wave solutions and discrete rogue wave solutions of KE equation were constructed by using DT and Taylor expansion in^18,19^. Ablowitz and Musslimani proposed the nonlocal modified Korteweg–de Vries (mKdV) equation and the nonlocal Sine–Gordon (SG) equation, and proved the integrability of these equations in^20^. Ji and Zhu obtained a series of different types of exact analytical solutions of nonlocal mKdV equations through constructing DT^21^, including complexiton solutions, rogue wave solutions, kink soliton solutions and anti-kink soliton solutions. Some bright soliton solutions, dark soliton solutions and breather solutions of the super integrable equation are presented with DT^22^. The non-autonomous multi-rogue wave solutions of the spin-1 coupled nonlinear Gross–Pitaevskii equation with different dispersion, higher-order nonlinear terms, gain (or loss) and external potential are considered in^23–25^. The multiple breather solutions and mixed solutions of the Kundu equation are constructed with generalized Darboux transformation method, which have the Lax pair of Kaup–Newell system in^26^.

The paper is organized as follows: in “Results”, we successfully solve the GNLS equation with DT, and obtain several new sets of exact solutions, including 1-soliton solutions, 2-soliton solutions and *n*-soliton solutions. In “Conclusions”, we select the non-zero seed solution and solve the GNLS equation by using the DT, and obtain the breather solutions of the GNLS equation. In “Methods”, we also use the DT and Taylor expansion to derive the rational wave solutions of the GNLS equation. Finally, we give some conclusions in “Rational wave solutions for GNLS Eq. (4)”.

## Results

### Soliton solutions of GNLS equation

It is well known that the standard nonlinear Schrödinger(NLS) equation

1 $$\begin{aligned} iu_{t}+\gamma u_{xx}+\sigma u|u|^2=0,\quad \quad \sigma = \pm 1 , \end{aligned}$$

is one of the most important integrable system among many branches of applied mathematics and physics, especially in optics, water wave and so on. The $u = u(x, t)$ is a complex smooth function of *x* and *t* , the subscripts denote partial derivatives and the parameter $\gamma$ is real constant in Eq. (1).

Fokas studied an integrable generalized nonlinear Schrödinger (GNLS) equation by means of bi-Hamiltonian operators

2 $$\begin{aligned} iu_t - vu_{tx} +\gamma u_{xx}+\sigma |u|^2(u+ivu_x)=0,\quad \quad \sigma = \pm 1, \end{aligned}$$

where $\gamma$ and *v* are real constants. In fact, Eq. (2) can be transformed into Eq. (1) when the parameter $v=0$. Lenells investigated Eq. (2) by the dressing method, and presented a new form of Eq. (2) as following

3 $$\begin{aligned} u_{tx} + \alpha \beta ^2 u - 2i\alpha \beta u_x - \alpha u_{xx} + \sigma i\alpha \beta ^2|u|^2u_x =0,\quad \quad \sigma =\pm 1, \end{aligned}$$

under the transformation of $u\rightarrow \beta \sqrt{\alpha }e^{i\beta x}u$, $\sigma =-\sigma$, where $\alpha =\frac{\gamma }{\nu }>0,\beta =\frac{1}{v}$.

Without losing generality, let $\sigma =-1$, then Eq. (3) will become the following form^27^:

4 $$\begin{aligned} u_{tx} + \alpha \beta ^2 u - 2i\alpha \beta u_x - \alpha u_{xx} - i\alpha \beta ^2|u|^2u_x =0, \end{aligned}$$

and the Lax pair of Eq. (4) is as following

5 $$\begin{aligned} \varphi _x= & {} U\varphi ,~~\varphi _t =V\varphi , \nonumber \\ U= & {} \left( \begin{array}{cc} -i \lambda ^2 &{}\lambda u_x\\ \lambda r_x&{} i \lambda ^2 \end{array}\right) ,\quad V=\left( \begin{array}{cc} -\frac{i\alpha \beta ^2}{2}ur - i \eta ^2 &{}\frac{i\alpha \beta ^2}{2\lambda }u + \alpha \lambda u_x\\ -\frac{i\alpha \beta ^2}{2\lambda }r + \alpha \lambda r_x&{}\frac{i\alpha \beta ^2}{2}ur + i \eta ^2 \end{array}\right) , \end{aligned}$$

where $$ \eta =\sqrt{\alpha }(\lambda - \frac{\beta }{2 \lambda }) ,r=-u^* $$, the $$ ``*''$$ denotes the complex conjugate and the vector $$ \varphi = ( \varphi _1, \varphi _2)^T$$ is an eigenfunction associated with $$ \lambda $$ and potential *u*, which consists of two complex functions $$ \varphi _1 = \varphi _1(x, t)$$ and $$ \varphi _2 = \varphi _2(x, t)$$. Trough direct calculations, we can verify that the integrability condition $$ U_t - V_x + [U, V] = 0 $$ exactly can be derived from Eq. (4), where $$ [U, V] = U V - V U $$.

From the above analysis, we could construct a *N*-fold Darboux matrix *T* for the GNLS equation (4), as follows

6 $$\begin{aligned} \widetilde{\varphi }_n=T \varphi _n,\quad T=\left( \begin{array}{cc} T_{11}&{}T_{12}\\ T_{21}&{} T_{22} \end{array}\right) . \end{aligned}$$

The lower forms are obtained by compatibility

7 $$\begin{aligned} \varphi _x= & {} \widetilde{U}\varphi ,~~\widetilde{U} =(T_x +TU)T^{-1}, \end{aligned}$$

8 $$\begin{aligned} \varphi _t= & {} \widetilde{V}\varphi ,~~\widetilde{V} =(T_t +TV)T^{-1}. \end{aligned}$$

If the $\widetilde{U},\widetilde{V}$ and *U*, *V* have the same types, the system (6) is called Darboux transformation of the GNLS equation. Let $\psi =(\psi _1,\psi _2)^{T}$, $\phi =(\phi _1,\phi _2)^{T}$ are two basic solutions of the systems (5), then we give the following linear algebraic systems:

9 $$\begin{aligned} {\left\{ \begin{array}{ll} \sum _{k=1}^{N}A_{k}\lambda _j^{2k}+\sum _{k=1}^{N}B_{k}\lambda _j^{2k-1} M_j^{(1)}=-1,\\ \sum _{k=1}^{N}-B_{k}^*\lambda _j^{2k-1}+\sum _{k=1}^{N}A_{k}^*\lambda _j^{2k}M_j^{(1)}=-M_j^{(1)}, \end{array}\right. } \end{aligned}$$

with

10 $$\begin{aligned} M_j^{(1)}=\frac{\psi _2+v_j^{(1)}\phi _2}{\psi _1+v_j^{(1)}\phi _1},\quad (1\le j \le 2N),\quad A_{k}^*=D_{k},~~~-B_{k}^*=C_{k}, \end{aligned}$$

where $\lambda _j$ and $v_j^{(k)}$ should choose appropriate parameters, thus the determinants of coefficients for Eq. (9) are nonzero. Hereby, we take a $2 \times 2$ matrix *T* as

11 $$\begin{aligned} {\left\{ \begin{array}{ll} T_{11}=1+\sum _{k=1}^{N}A_{k}\lambda ^{2k},~~T_{12}=\sum _{k=1}^{N}B_{k}\lambda ^{2k-1},\\ T_{21}=\sum _{k=1}^{N}-B_{k}^*\lambda ^{2k-1},~~T_{22}=1+\sum _{k=1}^{N}A_{k}^*\lambda ^{2k}, \end{array}\right. } \end{aligned}$$

where *N* is a natural number, the $A_{mn}^{ (i)} (m, n = 1, 2, i \ge 0)$ are some functions of *x* and *t*. Through calculations, we can obtain $\Delta T$ as following

12 $$\begin{aligned} \Delta T = \Pi _{j=1}^{2N}(\lambda -\lambda _j), \end{aligned}$$

which proves that $\lambda _j~(\lambda _j \ne 0)(j=1, 2, 3, \ldots , 2N)$ are 2*N* roots of $\Delta T$. Based on these conditions, we will proof that the $\widetilde{U}$ and $\widetilde{V}$ have the same structures as *U* and *V* respectively.

The matrix $\widetilde{U}$ defined by (7) has the same type as *U*, that is,

13 $$\begin{aligned} \widetilde{U}=\left( \begin{array}{cc} -i\lambda ^2&{}\lambda \widetilde{u}_x\\ \lambda \widetilde{r}_x&{}i\lambda ^2 \end{array}\right) , \end{aligned}$$

in which the transformation formula between old and new potentials are defined by

14 $$\begin{aligned} {\left\{ \begin{array}{ll}\widetilde{u}_x=u_x + B_{1x},\\ \widetilde{r}_x=r_x + C_{1x},\end{array}\right. } \end{aligned}$$

the transformations (14) are used to get a Darboux transformation of the spectral problem (7).

Let $$T^{-1}= \frac{T^{*}}{\Delta T}$$ and

15 $$\begin{aligned} (T_x+TU)T^{*}=\left( \begin{array}{cc} B_{11}(\lambda )&{}B_{12}(\lambda )\\ B_{21}(\lambda )&{} B_{22}(\lambda ) \end{array}\right) , \end{aligned}$$

it is easy to verify that $B_{sl} (1 \le s, l \le 2)$ is 2*N*-order or $2N+1$-order polynomial of $\lambda$.

Through some accurate calculations, $\lambda _j(1 \le j \le 2,)$ is the root of $B_{sl} (1 \le s, l \le 2)$. Thus, Eq. (15) has the following structure

16 $$\begin{aligned} (T_x+TU)T^{*}=(\Delta T)E(\lambda ), \end{aligned}$$

where

17 $$\begin{aligned} E(\lambda )=\left( \begin{array}{cc} E_{11}^{(2)}\lambda ^2+E_{11}^{(1)}\lambda +E_{11}^{(0)}&{}E_{12}^{(1)}\lambda +E_{12}^{(0)}\\ E_{21}^{(1)}\lambda +E_{21}^{(0)}&{} E_{22}^{(2)}\lambda ^2+E_{22}^{(1)}\lambda +E_{22}^{(0)} \end{array}\right) , \end{aligned}$$

and $E_{mn}^{(k)} (m, n = 1, 2, k = 0, 1)$ satisfy the functions without $\lambda$ . Equation (16) can be rewritten as

18 $$\begin{aligned} (T_x+TU)=E(\lambda )T. \end{aligned}$$

Through comparing the coefficients of $\lambda$ in Eq. (18), we can obtain

19 $$\begin{aligned} {\left\{ \begin{array}{ll} E_{11}^{(0)}=0,E_{11}^{(1)}=0,E_{11}^{(2)}=i, E_{12}^{(0)}=0,E_{12}^{(1)}=u_x+B_{1x}=\widetilde{u}_x,\\ E_{21}^{(0)}=0,E_{21}^{(1)}=r_x+C_{1x}=\widetilde{r}_x, E_{22}^{(0)}=0,E_{22}^{(1)}=0,E_{22}^{(2)}=i. \end{array}\right. } \end{aligned}$$

In this section, we assume that the new matrix $\widetilde{U}$ has the same type with *U*, which means that they have the same structures only *u*(*x*, *t*), *r*(*x*, *t*) of *U* transformed into $\widetilde{u}(x, t), \widetilde{r}(x, t)$ of $\widetilde{U}$. After careful calculation, we compare the ranks of $\lambda ^N$, and get the objective equations as following:

20 $$\begin{aligned} {\left\{ \begin{array}{ll}\widetilde{u}_x=u_x + B_{1x},\\ \widetilde{r}_x=r_x + C_{1x}, \end{array}\right. } \end{aligned}$$

from Eqs. (13) and (14), we know that $\widetilde{U} = E(\lambda )$. The proof is completed.

The matrix $\widetilde{V}$ defined by the second expression of (8) has the same form as *V*, in which the old potentials *u* and *r* are mapped into $\widetilde{u}$ and $\widetilde{r}$, that is,

21 $$\begin{aligned} \widetilde{V}=\left( \begin{array}{cc} -\frac{i\alpha \beta ^2}{2}\widetilde{u}\widetilde{r} - i \eta ^2 &{}\frac{i\alpha \beta ^2}{2\lambda }\widetilde{u} + \alpha \lambda \widetilde{u}_x\\ -\frac{i\alpha \beta ^2}{2\lambda }\widetilde{r} + \alpha \lambda \widetilde{r}_x&{}\frac{i\alpha \beta ^2}{2}\widetilde{u}\widetilde{r} + i \eta ^2 \end{array}\right) . \end{aligned}$$

We suppose the new matrix $\widetilde{V}$ also has the same form with *V*. If we obtain the similar relations between *u*(*x*, *t*), *r*(*x*, *t*) and $\widetilde{u}(x, t), \widetilde{r}(x, t)$ in Eq. (14), we can prove that the gauge transformations under *T* turn the Lax pairs *U*, *V* into new Lax pairs $\widetilde{U}, \widetilde{V}$ with the same types.

Let $$T^{-1}= \frac{T^{*}}{\Delta T}$$ and

22 $$\begin{aligned} (T_t+TV)T^{*}=\left( \begin{array}{cc} C_{11}(\lambda )&{}C_{12}(\lambda )\\ C_{21}(\lambda )&{} C_{22}(\lambda ) \end{array}\right) , \end{aligned}$$

it is easy to verify that $C_{sl} (1 \le s, l \le 2)$ is 2*N*-order or $2N+1$-order polynomial of $\lambda$. Through some accurate calculations, $\lambda _j (1 \le j \le 2)$ is the root of $C_{sl} (1 \le s, l \le 2)$. Thus, Eq. (22) has the following structure

23 $$\begin{aligned} (T_t+TV)T^{*}=(\Delta T)F(\lambda ), \end{aligned}$$

where

24 $$\begin{aligned} F(\lambda )=\left( \begin{array}{cc} F_{11}^{(2)}\lambda ^2+F_{11}^{(0)}+F_{11}^{(-2)}\lambda ^{-2}&{}F_{12}^{(1)}\lambda +F_{12}^{(-1)}\lambda ^{-1}\\ F_{21}^{(1)}\lambda +F_{21}^{(-1)}\lambda ^{-1}&{} F_{22}^{(2)}\lambda ^2+F_{22}^{(0)}F_{22}^{(-2)}\lambda ^{-2} \end{array}\right) , \end{aligned}$$

and $F_{mn}^{(k)} (m, n = 1, 2, k = 0, 1)$ satisfies the functions without $\lambda$. According to Eq. (23), the following equation is obtained

25 $$\begin{aligned} (T_t+TV)=F(\lambda )T. \end{aligned}$$

Through comparing the coefficients of $\lambda$ in Eq. (25), we get the objective equations as following:

26 $$\begin{aligned} {\left\{ \begin{array}{ll} F_{11}^{(-2)}=-\frac{i\alpha \beta ^{2}}{4},~F_{11}^{(2)}=-i\alpha ,~F_{11}^{(0)}=i\alpha \beta -\frac{i\alpha \beta ^{2}}{2}\widetilde{u}\widetilde{r},\\ F_{12}^{(-1)}=\frac{i\alpha \beta ^{2}}{2}\widetilde{u},~F_{12}^{(1)}=\frac{A_Nu_x\alpha +2i\alpha B_N}{D_N},\\ F_{21}^{(-1)}=-\frac{i\alpha \beta ^2}{2}\widetilde{r},~F_{21}^{(1)}=\frac{D_Nr_x\alpha -2i\alpha C_N}{A_N}, \\ F_{22}^{(-2)}=\frac{i\alpha \beta ^2}{4},~F_{22}^{(2)}=i\alpha ,~F_{22}^{(0)}=-i\alpha \beta +\frac{i\alpha \beta ^2}{2}\widetilde{u}\widetilde{r}. \end{array}\right. } \end{aligned}$$

In this section, we assume the new matrix $\widetilde{V}$ has the same type with *V*, which means they have the same structures only *u*(*x*, *t*), *r*(*x*, *t*) of *V* transformed into $\widetilde{u}(x, t), \widetilde{r}(x, t)$ of $\widetilde{V}$. From Eqs. (14) and (21), we know that $\widetilde{V}=F(\lambda )$ . The proof is completed.

We will give some novel explicit solutions of Eq. (4) by applying *N*-fold DT. Firstly, we give a seed solution $u = 0$ and substitute the solution into Eq. (5), it is easy to find two basic solutions for these equations:

27 $$\begin{aligned} \psi (\lambda )=\left( \begin{array}{c} e^{-i\lambda ^2x-i\eta ^2t+C_1}\\ 0 \end{array}\right) ,\quad \phi (\lambda )=\left( \begin{array}{c} 0\\ e^{i\lambda ^2x+i\eta ^2t+C_2} \end{array}\right) , \end{aligned}$$

by using Eqs. (8) and (25), we obtain

28 $$\begin{aligned} M_j^{(1)}={\frac{v_j^{(1)}e^{-i\lambda ^2x-i\eta ^2t+C_1}}{e^{i\lambda ^2x+i\eta ^2t+C_2}}}=e^{2(i\lambda _j^2x+i\eta ^2t+F_j)}, \end{aligned}$$

with $$\nu _j^{(i)}=e^{(2iF_{ji})}$$ $$(1\le i\le 2, 1\le j \le 2N)$$.

In order to derive the expression of *N*-order DT of Eq. (4) and obtain the matrix *T*

29 $$\begin{aligned} T=\left( \begin{array}{cc} 1+\sum _{k=1}^{N}A_{k}\lambda ^{2k}&{}\sum _{k=1}^{N}B_{k}\lambda ^{2k-1}\\ \sum _{k=1}^{N}-B_{k}^*\lambda ^{2k-1}&{}1+\sum _{k=1}^{N}A_{k}^*\lambda ^{2k}\end{array}\right) , \end{aligned}$$

and

30 $$\begin{aligned} {\left\{ \begin{array}{ll} \sum _{k=1}^{N}A_{k}\lambda _j^{2k}+\sum _{k=1}^{N}B_{k}\lambda _j^{2k-1} M_j^{(1)}+1=0,\\ \sum _{k=1}^{N}-B_{k}^*\lambda _j^{2k-1}+\sum _{k=1}^{N}A_{k}^*\lambda _j^{2k}M_j^{(1)}+M_j^{(1)}=0. \end{array}\right. } \end{aligned}$$

Solving Eq. (30) via the Gramer’s rule, we have

31 $$\begin{aligned} B_{N}=\frac{\Delta B_{N}}{\Delta },C_{N}=\frac{\Delta C_{N}}{\Delta } \end{aligned}$$

with

32 $$\begin{aligned} \Delta= & {} \left| \begin{array}{ccccccccc} \lambda _1^2&{}\lambda _1^4&{}\lambda _1^6&{}\ldots &{}\lambda _1^{2N}&{}M_1\lambda _1&{}M_1\lambda _1^{3}&{}\ldots &{}M_1\lambda _1^{(2N-1)}\\ \lambda _2^2&{}\lambda _2^4&{}\lambda _2^6&{}\ldots &{}\lambda _2^{2N}&{}M_2\lambda _2&{}M_2\lambda _2^{3}&{}\ldots &{}M_2\lambda _2^{(2N-1)}\\ \lambda _3^2&{}\lambda _3^4&{}\lambda _3^6&{}\ldots &{}\lambda _3^{2N}&{}M_3\lambda _3&{}M_3\lambda _3^{3}&{}\ldots &{}M_3\lambda _3^{(2N-1)}\\ \vdots &{}\vdots &{}\vdots &{}\ldots &{}\vdots &{}\vdots &{}\vdots &{}\ldots &{}\vdots \\ \lambda _{2N}^2&{}\lambda _{2N}^4&{}\lambda _{2N}^6&{}\ldots &{}\lambda _{2N}^{2N}&{}M_{2N}\lambda _{2N}&{}M_{2N}\lambda _{2N}^{3}&{}\ldots &{}M_{2N}\lambda _{2N}^{(2N-1)} \end{array}\right| ,\nonumber \\ \Delta B_{N}= & {} \left| \begin{array}{ccccccccc} \lambda _1^2&{}\lambda _1^4&{}\lambda _1^6&{}\ldots &{}\lambda _1^{2N}&{}M_1\lambda _1&{}M_1\lambda _1^{3}&{}\ldots &{}-1\\ \lambda _2^2&{}\lambda _2^4&{}\lambda _2^6&{}\ldots &{}\lambda _2^{2N}&{}M_2\lambda _2&{}M_2\lambda _2^{3}&{}\ldots &{}-1\\ \lambda _3^2&{}\lambda _3^4&{}\lambda _3^6&{}\ldots &{}\lambda _3^{2N}&{}M_3\lambda _3&{}M_3\lambda _3^{3}&{}\ldots &{}-1\\ \vdots &{}\vdots &{}\vdots &{}\ldots &{}\vdots &{}\vdots &{}\vdots &{}\ldots &{}\vdots \\ \lambda _{2N}^2&{}\lambda _{2N}^4&{}\lambda _{2N}^6&{}\ldots &{}\lambda _{2N}^{2N}&{}M_{2N}\lambda _{2N}&{}M_{2N}\lambda _{2N}^{3}&{}\ldots &{}-1 \end{array}\right| ,\nonumber \\ \Delta C_{N}= & {} \left| \begin{array}{ccccccccc} \lambda _1&{}\lambda _1^3&{}\lambda _1^5&{}\ldots &{}-M_1&{}M_1\lambda _1^2&{}M_1\lambda _1^{4}&{}\ldots &{}M_1\lambda _1^{2N}\\ \lambda _2&{}\lambda _2^3&{}\lambda _2^5&{}\ldots &{}-M_2&{}M_2\lambda _2^2&{}M_2\lambda _2^{4}&{}\ldots &{}M_2\lambda _2^{2N}\\ \lambda _3&{}\lambda _3^3&{}\lambda _3^5&{}\ldots &{}-M_3&{}M_3\lambda _3^2&{}M_3\lambda _3^{4}&{}\ldots &{}M_3\lambda _3^{2N}\\ \vdots &{}\vdots &{}\vdots &{}\ldots &{}\vdots &{}\vdots &{}\vdots &{}\ldots &{}\vdots \\ \lambda _{2N}&{}\lambda _{2N}^3&{}\lambda _{2N}^5&{}\ldots &{}-M_{2N}&{}M_{2N}\lambda _{2N}^2&{}M_{2N}\lambda _{2N}^{4}&{}\ldots &{}M_{2N}\lambda _{2N}^{2N} \end{array}\right| . \end{aligned}$$

**Figure 1:**
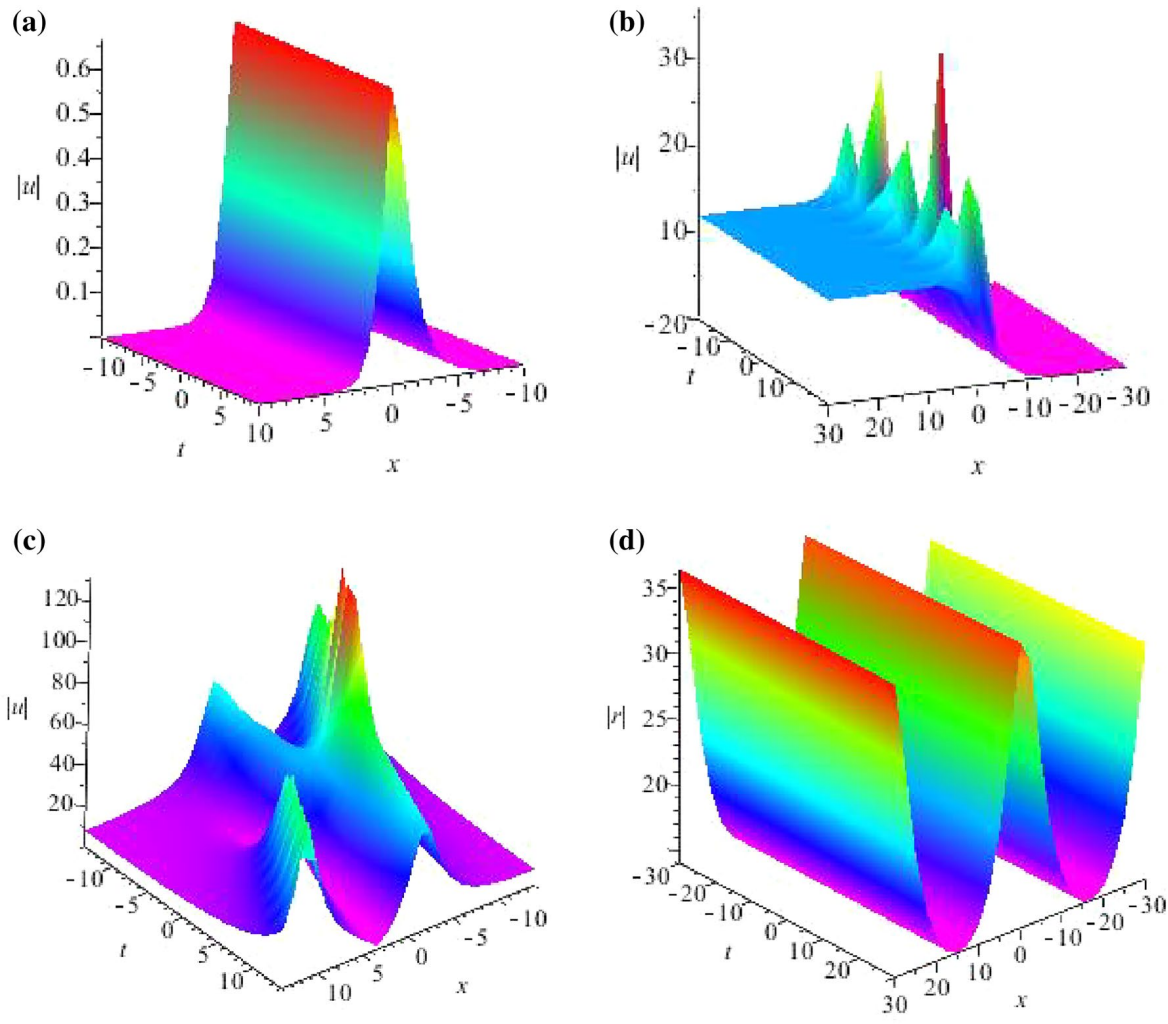
Profiles of intensity distribution (**a**) $|\widetilde{u}(x,t)|$ of Eq. (34) with parameters $\lambda _1=1-0.8i,\lambda _2=0.6+0.4i,\alpha =0.0004,\beta =1,F_1=0.4+i,F_2=0.3+0.6i$; (**b**) $|\widetilde{u}(x,t)|$ of Eq. (34) with parameters $\lambda _1=0.2i,\lambda _2=0.1,\alpha =0.4,\beta =0.2,F_1=0.01,F_2=0.02$; (**c**) $|\widetilde{u}(x,t)|$ of Eq. (36) with parameters $\lambda _1=0.2,\lambda _2=0.3+0.2i,\lambda _3=0.3,\lambda _4=0.3-0.2i,\alpha =0.2,\beta =0.3,F_1=0.2+0.2i,F_2=0.3-0.2i,F_3=0.3+0.2i,F_4=0.3-0.2i$; (**d**) $|\widetilde{r}(x,t)|$ of Eq. (36) with parameters $\lambda _1=0.5,\lambda _2=0.2,\lambda _3=0.5,\lambda _4=0.3,\alpha =0.004,\beta =0.2,F_1=0.5+0.2i,F_2=0.5-0.2i,F_3=0.3+0.1i,F_4=0.3-0.1i$.

Using Eqs. (6), (20) and (31), we can derive the new formulas of *N*-soliton solutions for GNLS equation

33 $$\begin{aligned} {\left\{ \begin{array}{ll} \widetilde{u}(x,t)=\frac{\Delta B_{N}}{\Delta },\\ \\ \widetilde{r}(x,t)=\frac{\Delta C_{N}}{\Delta }, \end{array}\right. } \end{aligned}$$

in order to understand solutions (33), we consider $N = 1, 2$ separately and plot their structure figures in Fig. 1a,b.

(I) We take $N = 1$ with $\lambda =\lambda _j (j=1,2)$. Solving Eq. (9), we can yield the 1-soliton solutions of the GNLS equation (4) as following:
  34 $$\begin{aligned} \widetilde{u}(x,t)=\frac{\Delta B_{1}}{\Delta },\quad \widetilde{r}(x,t)=-\widetilde{u}^*(x,t), \end{aligned}$$
  with
  35 $$\begin{aligned} \Delta= & {} \left| \begin{array}{cc} \lambda _1^2&{}e^{2(i\lambda _1x+i\eta ^2t+F_1)}\lambda _1\\ \lambda _2^2&{}e^{2(i\lambda _2x+i\eta ^2t+F_2)}\lambda _2 \end{array}\right| ,\quad \Delta B_1=\left| \begin{array}{cc} \lambda _1^2&{}-1\\ \lambda _2^2&{}-1\end{array}\right| , \nonumber \\ \Delta C_1= & {} \left| \begin{array}{cc} -e^{2(i\lambda _1x+i\eta ^2t+F_1)}&{}\lambda _1^2e^{2(i\lambda _1x+i\eta ^2t+F_1)}\\ -e^{2(i\lambda _2x+i\eta ^2t+F_2)}&{}\lambda _2^2e^{2(i\lambda _2x+i\eta ^2t+F_2)} \end{array}\right| . \end{aligned}$$
(II) We take $N = 2$ in the *N*-times DT with $\lambda = \lambda _j (j=1, 2, 3, 4)$. The linear algebraic system (9) leads to the 2-soliton solutions of GNLS (4) as following:
  36 $$\begin{aligned} \widetilde{u}(x,t)=\frac{\Delta B_{2}}{\Delta },~~~ \widetilde{r}(x,t)=-\widetilde{u}^*(x,t), \end{aligned}$$
  with
  37 $$\begin{aligned} \Delta= & {} \left| \begin{array}{cccc} \lambda _1^2&{}\lambda _1^4&{}e^{2(i\lambda _1x+i\eta ^2t+F_1)}\lambda _1&{}e^{2(i\lambda _1x+i\eta ^2t+F_1)}\lambda _1^3\\ \lambda _2^2&{}\lambda _2^4&{}e^{2(i\lambda _2x+i\eta ^2t+F_2)}\lambda _2&{}e^{2(i\lambda _2x+i\eta ^2t+F_2)}\lambda _2^3\\ \lambda _3^2&{}\lambda _3^4&{}e^{2(i\lambda _3x+i\eta ^2t+F_3)}\lambda _3&{}e^{2(i\lambda _3x+i\eta ^2t+F_3)}\lambda _3^3\\ \lambda _4^2&{}\lambda _4^4&{}e^{2(i\lambda _4x+i\eta ^2t+F_4)}\lambda _4&{}e^{2(i\lambda _4x+i\eta ^2t+F_4)}\lambda _4^3 \end{array}\right| ,\quad \Delta B_2=\left| \begin{array}{cccc} \lambda _1^2&{}\lambda _1^4&{}e^{2(i\lambda _1x+i\eta ^2t+F_1)}\lambda _1&{}-1\\ \lambda _2^2&{}\lambda _2^4&{}e^{2(i\lambda _2x+i\eta ^2t+F_2)}\lambda _2&{}-1\\ \lambda _3^2&{}\lambda _3^4&{}e^{2(i\lambda _3x+i\eta ^2t+F_3)}\lambda _3&{}-1\\ \lambda _4^2&{}\lambda _4^4&{}e^{2(i\lambda _4x+i\eta ^2t+F_4)}\lambda _4&{}-1\end{array}\right| , \nonumber \\ \Delta C_2= & {} \left| \begin{array}{cccc} \lambda _1&{}-e^{2(i\lambda _1x+i\eta ^2t+F_1)}&{}\lambda _1^2e^{2(i\lambda _1x+i\eta ^2t+F_1)}&{}\lambda _1^4e^{2(i\lambda _1x+i\eta ^2t+F_1)}\\ \lambda _2&{}-e^{2(i\lambda _2x+i\eta ^2t+F_2)}&{}\lambda _2^2e^{2(i\lambda _2x+i\eta ^2t+F_2)}&{}\lambda _2^4e^{2(i\lambda _2x+i\eta ^2t+F_2)}\\ \lambda _3&{}-e^{2(i\lambda _3x+i\eta ^2t+F_3)}&{}\lambda _3^2e^{2(i\lambda _3x+i\eta ^2t+F_3)}&{}\lambda _3^4e^{2(i\lambda _3x+i\eta ^2t+F_3)}\\ \lambda _4&{}-e^{2(i\lambda _4x+i\eta ^2t+F_4)}&{}\lambda _4^2e^{2(i\lambda _4x+i\eta ^2t+F_4)}&{}\lambda _4^4e^{2(i\lambda _4x+i\eta ^2t+F_4)} \end{array}\right| . \end{aligned}$$

In order to understand solutions (36), we consider $N=2$ and plot their structure figures in Fig. 1c,d.

## Conclusions

The integrable GNLS equation can describe the propagation of nonlinear light pulses in optical fibers, the high-order nonlinear effects are taken into consideration. In this paper, we investigate the exact solutions (including soliton solutions, breather solutions, and rational wave solutions) of a GNLS equation via DT method. And the 1-solitons, 2-solitons and *N*-soliton solutions of the GNLS equation are obtained via constructing special Lax pairs. And we choose different seed solutions and obtain three kinds of solutions. Based on these obtained solutions, we consider the elastic interactions and dynamics between two solitons for the GNLS equation.

## Methods

### Breather solutions for GNLS equation (4)

Now we choose there kinds of seed solutions of (4) as follows:

38 $$\begin{aligned} u= & {} c_0e^{i\sigma \gamma _0^2x},~~c_0=\frac{\beta +\sigma \gamma _0^2}{\beta \gamma _0}, \end{aligned}$$

39 $$\begin{aligned} u= & {} \frac{\omega _0}{\beta \gamma _0}e^{-i(\gamma _0^2x+\delta _0t)},~~\delta _0=\alpha [(\beta +\sigma \gamma _0^2)^2-\omega _0^2]\gamma _0^{-2}, \end{aligned}$$

and

40 $$\begin{aligned} u=e^{i\theta },\quad \theta =ax+bt, \end{aligned}$$

where $\gamma _0$, $\omega _0$, *a* and *b* are arbitrary constants.

**Case 1:** We give a seed solution $u=c_0e^{i\sigma \gamma _0^2x}$ with $c_0=\frac{\beta +\sigma \gamma _0^2}{\beta \gamma _0}$. According to Eq. (5), we can yield the following systems

41 $$\begin{aligned} {\left\{ \begin{array}{ll} -i\lambda ^2\psi _1+i\sigma \gamma _0^2c_0\lambda e^{i\sigma \gamma _0^2x}\psi _2=\psi _{1x},\\ i\sigma \gamma _0^2c_0\lambda e^{-i\sigma \gamma _0^2x}\psi _1+i\lambda ^2\psi _2=\psi _{2x}, \end{array}\right. } \end{aligned}$$

without loss of generality, we assume that $\sigma =-1$, $\psi _1=\alpha e^{px}$, $\psi _2=\gamma e^{px - i\sigma \gamma _0^2x}$, then Eq. (41) is solved by

42 $$\begin{aligned} {\left\{ \begin{array}{ll} p=\frac{(i\lambda ^2\alpha -i\alpha \gamma _0^2-i\lambda ^2) \pm \sqrt{(i\alpha \gamma _0^2-i\lambda ^2\alpha +i\lambda ^2)^2 -4\alpha (\lambda ^4-\gamma _0^2\lambda ^2+\gamma _0^4c_0^2\alpha \lambda ^2)}}{2\alpha },\\ \gamma =\frac{-i\gamma _0^2c_0\lambda \alpha }{\beta +i\gamma _0^2-i\lambda ^2}. \end{array}\right. } \end{aligned}$$

Based on Eq. (5), we obtain

43 $$\begin{aligned} {\left\{ \begin{array}{ll} \left( \frac{i\alpha \beta ^2}{2}c_0^2e^{-2i\gamma _0^2x}-i\eta ^2\right) \psi _1 +e^{-i\gamma _0^2x}\left( \frac{i\alpha \beta ^2c_0}{2\lambda }-i\alpha c_0\gamma _0^2\lambda \right) \psi _2=\psi _{1t},\\ e^{-i\gamma _0^2x}\left( \frac{i\alpha \beta ^2c_0}{2\lambda }+i\alpha c_0\gamma _0^2\lambda \right) \psi _1+\left( -\frac{i\alpha \beta ^2c_0^2}{2}e^{-2i\gamma _0^2x}+i\eta ^2\right) \psi _2=\psi _{2t}, \end{array}\right. } \end{aligned}$$

we can derive the following system form Eq. (43)

44 $$\begin{aligned} {\left\{ \begin{array}{ll} \left( \frac{i\alpha \beta ^2}{2}c_0^2e^{-2i\gamma _0^2x}-i\eta ^2-\lambda _{11}\right) a + \left( e^{-i\gamma _0^2x}\frac{i\alpha \beta ^2c_0}{2\lambda }-e^{-i\gamma _0^2x}i\alpha c_0\gamma _0^2\lambda \right) b = 0,\\ \left( e^{-i\gamma _0^2x}\frac{i\alpha \beta ^2c_0}{2\lambda }+e^{i\gamma _0^2x}i\alpha c_0\gamma _0^2\lambda \right) a + \left( -\frac{i\alpha \beta ^2c_0^2}{2}e^{-2i\gamma _0^2x}+i\eta ^2-\lambda _{12}\right) b = 0. \end{array}\right. } \end{aligned}$$

We obtain that

$$\begin{aligned} \lambda _{11}=\sqrt{\alpha \beta ^2c_0^2\eta ^2-\eta ^4-\frac{\alpha ^2\beta ^4c_0^2}{4\lambda ^2}},\quad \lambda _{12}=-\sqrt{\alpha \beta ^2c_0^2\eta ^2-\eta ^4-\frac{\alpha ^2\beta ^4c_0^2}{4\lambda ^2}}, \end{aligned}$$

with

$$\begin{aligned} a=\left( \frac{i\alpha c_0\gamma _0^2\lambda e^{-i\gamma _0^2x}-\frac{i\alpha \beta ^2c_0}{2\lambda }e^{-i\gamma _0^2x}}{\frac{i\alpha \beta ^2c_0^2}{2}-i\eta ^2-\lambda _{11}}\right) b, \end{aligned}$$

substituting the above solutions and Eq. (44) into Eq. (5), it is easy to find two basic solutions for these equations:

45 $$\begin{aligned} \left( \begin{array}{c} \psi _1\\ \psi _2 \end{array}\right) =C_1e^{\lambda _{11}t}\left( \begin{array}{c} \frac{i\alpha c_0\gamma _0^2\lambda -\frac{-i\alpha \beta ^2c_0^2}{2}e^{-i\gamma _0^2x}}{\frac{i\alpha \beta ^2c_0^2}{2}e^{-2i\gamma _0^2x}-i\eta ^2-\lambda _{11}}\\ 1 \end{array}\right) + C_2e^{\lambda _{12}t}\left( \begin{array}{c}1\\ \frac{\frac{i\alpha \beta ^2c_0^2}{2}e^{-2i\gamma _0^2x}+i\eta ^2+\lambda _{12}}{e^{-2i\gamma _0^2x}\frac{i\alpha \beta ^2c_0}{2\lambda }-e^{-i\gamma _0^2x}i\alpha c_0\gamma _0^2\lambda } \end{array}\right) . \end{aligned}$$

It is easy to find two basic solutions for Eqs. (42) and (45):

46 $$\begin{aligned} \left( \begin{array}{c} \psi \\ \phi \end{array}\right) =\left( \begin{array}{c}\alpha C_1e^{\lambda _{11}t+p x}\left( \frac{i\alpha c_0\gamma _0^2\lambda e^{-i\gamma _0^2x}-\frac{i\alpha \beta ^2c_0}{2\lambda }e^{-i\gamma _0^2x}}{\frac{i\alpha \beta ^2c_0^2}{2}-i\eta ^2-\lambda _{11}}\right) +\alpha C_2e^{\lambda _{12}t+px} \\ \gamma C_1e^{\lambda _{11}t+p x+i\sigma \gamma _0^2x}+\gamma C_2e^{\lambda _{12}t+p x+i\sigma \gamma _0^2x}\left( \frac{e^{i\gamma _0^2x}\frac{i\alpha \beta ^2c_0}{2\lambda }-e^{i\gamma _0^2x}i\alpha c_0\gamma _0^2\lambda }{\frac{i\alpha \beta ^2c_0^2}{2}-i\eta ^2+\lambda _{12}}\right) \end{array}\right) , \end{aligned}$$

we can obtain by using Eq. (10),

47 $$\begin{aligned} M_j=\frac{C_1\gamma e^{F_j+i\gamma _0^2x+\lambda _{11}t}+\frac{C_2\gamma e^{F_j+2i\gamma _0^2x+\lambda _{12}t}(i\alpha \beta ^2c_0-2i\alpha c_0\lambda ^2\gamma _0^2)}{\lambda (i\alpha \beta ^2c_0^2-2i\eta ^2+2\lambda _{12})}}{C_2\alpha e^{\lambda _{12}t}+\frac{C_1\alpha e^{\lambda _{11}t-i\gamma _0^2x}(2i\alpha \lambda ^2c_0\gamma _0^2-i\alpha \beta ^2c_0)}{\lambda (i\alpha \beta ^2c_0^2-2i\eta ^2-2\lambda _{11})}},\quad 1\le j \le 2N, \end{aligned}$$

with $$\nu _j^{(i)}=e^{F_j}$$ $$(1\le i\le 2, 1\le j \le 2N)$$.

(I) We take $N = 1$ with $\lambda =\lambda _j$ (j = 1, 2). We can yield the 1-soliton solutions of the GNLS equation (4) from Eq. (9) as following:
  48 $$\begin{aligned} \widetilde{u}(x,t)=c_0e^{i\sigma \gamma _0^2x}+\frac{\Delta B_{1}}{\Delta },\quad \widetilde{r}(x,t)=-\widetilde{u}^*(x,t), \end{aligned}$$
  with
  49 $$\begin{aligned} \Delta =\left| \begin{array}{cc} \lambda _1^2&{}M_1\lambda _1\\ \lambda _2^2&{}M_2\lambda _2 \end{array}\right| ,~ \Delta B_1=\left| \begin{array}{cc} \lambda _1^2&{}-1\\ \lambda _2^2&{}-1\end{array}\right| ,~\Delta C_1=\left| \begin{array}{cc} -M_1&{}\lambda _1^2M_1\\ -M_2&{}\lambda _2^2M_2 \end{array}\right| . \end{aligned}$$
(II) We take $N = 2$ in the *N*-times DT with $\lambda = \lambda _j (j=1, 2, 3, 4)$. The linear algebraic system (9) leads to 2-soliton solutions of GNLS Eq. (4) as following:
  50 $$\begin{aligned} \widetilde{u}(x,t)=c_0e^{i\sigma \gamma _0^2x}+\frac{\Delta B_{2}}{\Delta },\quad \widetilde{r}(x,t)=-\widetilde{u}^*(x,t), \end{aligned}$$
  with
  51 $$\begin{aligned} \Delta =\left| \begin{array}{cccc} \lambda _1^2&{}\lambda _1^4&{}M_1\lambda _1&{}M_1\lambda _1^3\\ \lambda _2^2&{}\lambda _2^4&{}M_2\lambda _2&{}M_2\lambda _2^3\\ \lambda _3^2&{}\lambda _3^4&{}M_3\lambda _3&{}M_3\lambda _3^3\\ \lambda _4^2&{}\lambda _4^4&{}M_4\lambda _4&{}M_4\lambda _4^3 \end{array}\right| ,~ \Delta B_2=\left| \begin{array}{cccc} \lambda _1^2&{}\lambda _1^4&{}M_1\lambda _1&{}-1\\ \lambda _2^2&{}\lambda _2^4&{}M_2\lambda _2&{}-1\\ \lambda _3^2&{}\lambda _3^4&{}M_3\lambda _3&{}-1\\ \lambda _4^2&{}\lambda _4^4&{}M_4\lambda _4&{}-1\end{array}\right| , ~ \Delta C_2=\left| \begin{array}{cccc} \lambda _1&{}-M_1&{}\lambda _1^2M_1&{}\lambda _1^4M_1\\ \lambda _2&{}-M_2&{}\lambda _2^2M_2&{}\lambda _2^4M_2\\ \lambda _3&{}-M_3&{}\lambda _3^2M_3&{}\lambda _3^4M_3\\ \lambda _4&{}-M_4&{}\lambda _4^2M_4&{}\lambda _4^4M_4 \end{array}\right| . \end{aligned}$$

**Figure 2:**
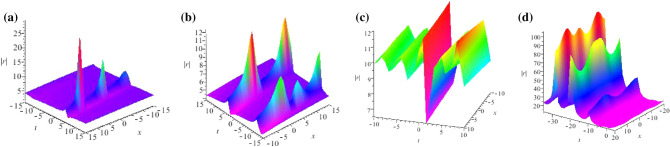
Profiles of intensity distribution (**a**) $|\widetilde{r}(x,t)|$ of Eq. (48) with parameters $\lambda _1=0.2+0.3i,\lambda _2=0.2-0.3i,\alpha =0.2,\beta =0.3,\gamma _0=0.2,\sigma =-1,F_1=0.3,F_2=0.2$; (**b**) $|\widetilde{r}(x,t)|$ of Eq. (48) with parameters $\lambda _1=0.3i,\lambda _2=0.2-0.4i,\alpha =0.2,\beta =0.3,\gamma _0=0.2,\sigma =-1,F_1=0.3,F_2=0.2$; (**c**) $|\widetilde{r}(x,t)|$ of Eq. (50) with parameters $\lambda _1=-0.3i,\lambda _2=0.2+0.3i,\lambda _3=0.1-0.3i,\lambda _4=0.4i,\alpha =0.6,\beta =0.2,\gamma _0=0.1,\sigma =-1,F_1=0.3,F_2=0.2,F_3=0.4,F_4=0.1$; (**d**) $|\widetilde{r}(x,t)|$ of Eq. (50) with parameters $\lambda _1=0.1i,\lambda _2=0.2-0.4i,\lambda _3=0.3i,\lambda _4=0.2i,\alpha =0.2,\beta =0.3,\gamma _0=0.5,\sigma =-1,F_1=0.3,F_2=0.2,F_3=0.4,F_4=0.1$.

Some periodic and breather solutions for GNLS equation (4) are shown, we consider $N=2$ and plot their structure figures in Fig. 2.

**Case 2:** We consider a solution $u=\frac{\omega _0}{\beta \gamma _0}e^{-i(\gamma _0^2x+\delta _0t)}$ with $\delta _0=\alpha [(\beta +\sigma \gamma _0^2)^2-\omega _0^2]\gamma _0^{-2}$. Based on Eq. (5), we can yield the following systems

52 $$\begin{aligned} {\left\{ \begin{array}{ll} -i\lambda \psi _1-\frac{i\omega _0\gamma _0^2}{\beta \gamma _0}e^{-i(\gamma _0^2x+\delta _0t)}\psi _2=\psi _{1x},\\ -\frac{i\omega _0\gamma _0^2}{\beta \gamma _0}e^{i(\gamma _0x+\delta _0t)}\psi _1+i\lambda \psi _2=\psi _{2x}, \end{array}\right. } \end{aligned}$$

without loss of generality, we assume that $\sigma =-1$, $\psi _1=\alpha _1 e^{\beta _1 x}$, $\psi _2=\gamma _1 e^{\beta _1 x + i(\gamma _0^2x+\delta _0t)}$, then Eq. (52) is solved by

53 $$\begin{aligned} {\left\{ \begin{array}{ll} \beta _1=\frac{-i\gamma _0^2\pm \sqrt{\Delta _1}}{2\beta ^2},\\ \alpha _1=\frac{-i\omega _0\gamma _0\gamma _1}{\beta (\beta _1+i\lambda )}, \end{array}\right. } \end{aligned}$$

we can obtain $\Delta _1=-\gamma _0^4\beta ^4-4\beta ^2(\omega _0^2\gamma _0^2+\lambda ^2\beta ^2-\lambda \gamma _0^2\beta ^2)$. By using Eq. (5), we obtain

54 $$\begin{aligned} {\left\{ \begin{array}{ll} \left( \frac{i\alpha \omega _0^2}{2\gamma _0^2}-i\eta ^2\right) \psi _1+\left( \frac{i\alpha \beta \omega _0}{2\lambda \gamma _0}e^{-i(\gamma _0^2x+\delta _0t)}-\frac{i\alpha \lambda \gamma _0\omega _0}{\beta }e^{-i(\gamma _0^2x+\delta _0t)}\right) \psi _2=\psi _{1t},\\ \left( \frac{i\alpha \beta \omega _0}{2\lambda \gamma _0}-\frac{i\alpha \lambda \gamma _0\omega _0}{\beta }\right) e^{i(\gamma _0^2x+\delta _0t)}\psi _1+\left( -\frac{i\alpha \omega _0^2}{2\gamma _0^2}+i\eta ^2\right) \psi _2=\psi _{2t}, \end{array}\right. } \end{aligned}$$

without loss of generality, we assume that $\psi _1=a_1 e^{c t}$, $\psi _2=b_1 e^{ct + i(\gamma _0^2x+\delta _0t)}$, then Eq. (54) is solved by

55 $$\begin{aligned} {\left\{ \begin{array}{ll} c=\frac{i\alpha \lambda \beta \omega _0^2-2i\lambda \beta \eta ^2\gamma _0^2+i\alpha \beta ^2\omega _0\gamma _0-2i\alpha \lambda ^2\gamma _0^3\omega _0}{2\lambda \beta \gamma _0^2a_1},\\ b_1=\frac{a_1(i\alpha \beta ^2\gamma _0\omega _0-2i\alpha \lambda ^2\gamma _0^3\omega _0-i\alpha \lambda \beta \omega _0^2+2i\eta ^2\lambda \beta \gamma _0^2)}{i\alpha \lambda \beta \omega _0^2-2i\lambda \beta \eta ^2\gamma _0^2+i\alpha \beta ^2\omega _0\gamma _0-2i\alpha \lambda ^2\gamma _0^3\omega _0+2i\lambda \beta \gamma _0^2\delta _0a_1}. \end{array}\right. } \end{aligned}$$

It is easy to find two basic solutions for Eqs. (53) and (55) as following

56 $$\begin{aligned} \left( \begin{array}{c} \psi \\ \phi \end{array}\right) =\left( \begin{array}{c} C_3e^{\beta _1x+ct} \\ C_4e^{\beta _1x+dt+2i(\gamma _0^2x+\delta _0t)} \end{array}\right) , \end{aligned}$$

we can derive by using Eq. (10),

57 $$\begin{aligned} M_j=\frac{e^{F_j}e^{dt+2i(\gamma _0^2x+\delta _0t)}}{e^{ct}},~~~1\le j \le 2N, \end{aligned}$$

with $$\nu _j^{(i)}=e^{F_j}$$ $$(1\le i\le 2, 1\le j \le 2N)$$.

(I) We take $N = 1$ with $\lambda =\lambda _j$ (j = 1, 2), and yield the 1-soliton solutions of the GNLS equation (4) as following:
  58 $$\begin{aligned} \widetilde{u}(x,t)=\frac{\omega _0}{\beta \gamma _0}e^{-i(\gamma _0^2x+\delta _0t)}+\frac{\Delta B_{1}}{\Delta },\quad \widetilde{r}(x,t)=-\widetilde{u}^*(x,t), \end{aligned}$$
  with
  59 $$\begin{aligned} \Delta =\left| \begin{array}{cc} \lambda _1^2&{}M_1\lambda _1\\ \lambda _2^2&{}M_2\lambda _2 \end{array}\right| ,~ \Delta B_1=\left| \begin{array}{cc} \lambda _1^2&{}-1\\ \lambda _2^2&{}-1\end{array}\right| ,~\Delta C_1=\left| \begin{array}{cc} -M_1&{}\lambda _1^2M_1\\ -M_2&{}\lambda _2^2M_2 \end{array}\right| . \end{aligned}$$
(II) We take $N = 2$ in the *N*-times DT with $\lambda = \lambda _j (j=1, 2, 3, 4)$. The linear algebraic system (9) leads to the 2-soliton solutions of GNLS Eq. (4) as following:
  60 $$\begin{aligned} \widetilde{u}(x,t)=\frac{\omega _0}{\beta \gamma _0}e^{-i(\gamma _0^2x+\delta _0t)}+\frac{\Delta B_{2}}{\Delta },\quad \widetilde{r}(x,t)=-\widetilde{u}^*(x,t), \end{aligned}$$
  with
  61 $$\begin{aligned} \Delta =\left| \begin{array}{cccc} \lambda _1^2&{}\lambda _1^4&{}M_1\lambda _1&{}M_1\lambda _1^3\\ \lambda _2^2&{}\lambda _2^4&{}M_2\lambda _2&{}M_2\lambda _2^3\\ \lambda _3^2&{}\lambda _3^4&{}M_3\lambda _3&{}M_3\lambda _3^3\\ \lambda _4^2&{}\lambda _4^4&{}M_4\lambda _4&{}M_4\lambda _4^3 \end{array}\right| , \Delta B_2=\left| \begin{array}{cccc} \lambda _1^2&{}\lambda _1^4&{}M_1\lambda _1&{}-1\\ \lambda _2^2&{}\lambda _2^4&{}M_2\lambda _2&{}-1\\ \lambda _3^2&{}\lambda _3^4&{}M_3\lambda _3&{}-1\\ \lambda _4^2&{}\lambda _4^4&{}M_4\lambda _4&{}-1\end{array}\right| , \Delta C_2=\left| \begin{array}{cccc} \lambda _1&{}-M_1&{}\lambda _1^2M_1&{}\lambda _1^4M_1\\ \lambda _2&{}-M_2&{}\lambda _2^2M_2&{}\lambda _2^4M_2\\ \lambda _3&{}-M_3&{}\lambda _3^2M_3&{}\lambda _3^4M_3\\ \lambda _4&{}-M_4&{}\lambda _4^2M_4&{}\lambda _4^4M_4 \end{array}\right| . \end{aligned}$$

**Figure 3:**
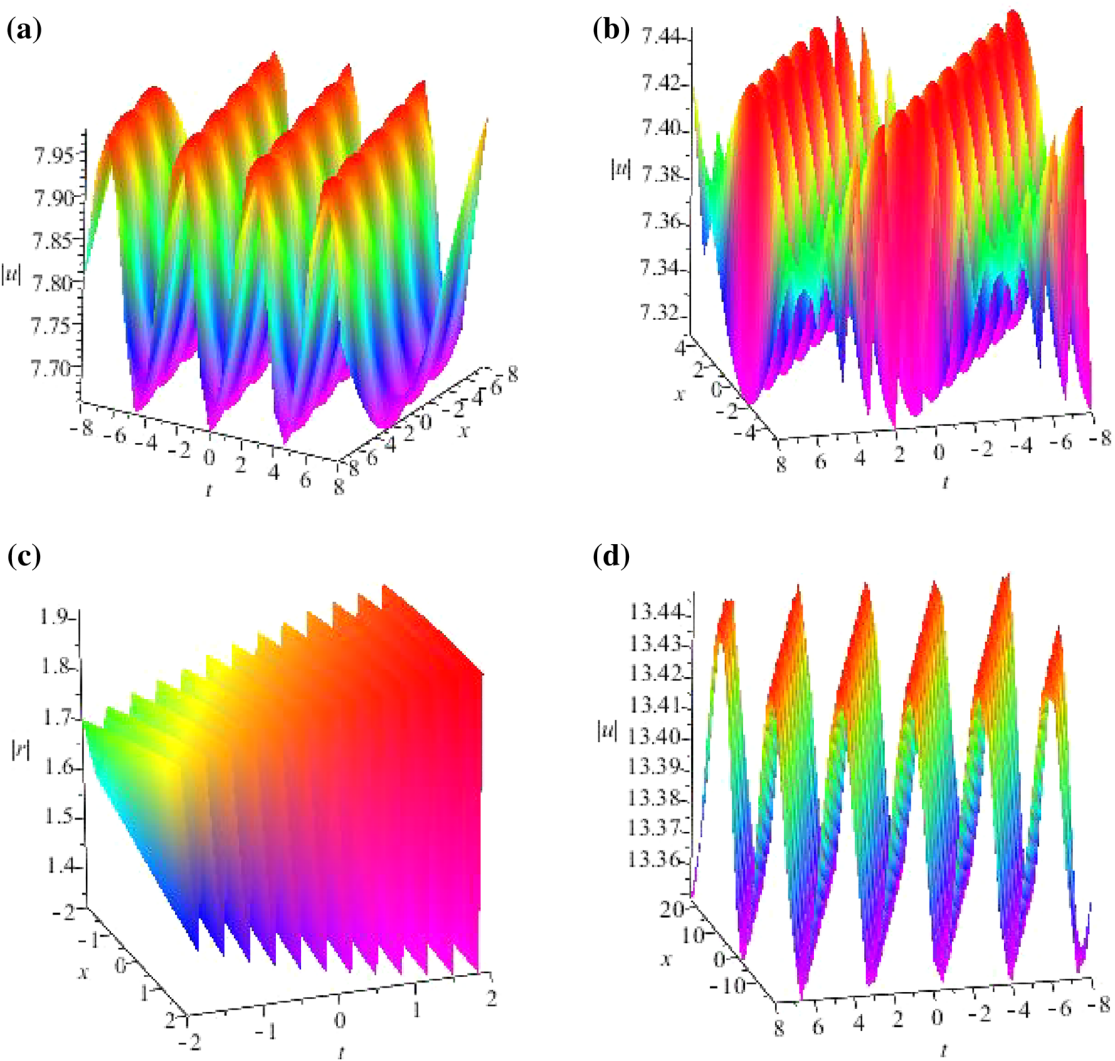
Profiles of intensity distribution (**a**) $|\widetilde{u}(x,t)|$ of Eq. (58) with parameters $\lambda _1=0.2,\lambda _2=0.3,\alpha =0.3,\beta =5,\gamma _0=0.5,\sigma =-1,\omega _0=0.4,F_1=0.3,F_2=0.4$; (**b**) $|\widetilde{u}(x,t)|$ of Eq. (58) with parameters $\lambda _1=0.3+0.2i,\lambda _2=0.3-0.2i,\alpha =0.4,\beta =5,\gamma _0=0.6,\sigma =-1,\omega _0=0.2,F_1=0.2+0.3i,F_2=0.2-0.3i$; (**c**) $|\widetilde{r}(x,t)|$ of Eq. (60) with parameters $\lambda _1=0.3i,\lambda _2=-0.2i,\lambda _3=0.4i,\lambda _4=-0.5i,\alpha =0.4,\beta =5,\gamma _0=0.2,\sigma =-1,\omega _0=0.3,F_1=0.3i,F_2=-0.3i,F_1=0.5i,F_1=-0.5i$; (**d**) $|\widetilde{u}(x,t)|$ of Eq. (60) with parameters $\lambda _1=0.3,\lambda _2=-0.2,\lambda _3=0.4,\lambda _4=0.5,\alpha =0.4,\beta =8,\gamma _0=0.5,\sigma =-1,\omega _0=0.2,F_1=0.3i,F_2=-0.3i,F_3=0.5i,F_4=-0.5i$.

Some periodic solutions for GNLS equation (4) with seed $u=\frac{\omega _0}{\beta \gamma _0}e^{-i(\gamma _0^2x+\delta _0t)}$ are shown, we consider $N=2$ and plot their structure figures in Fig. 3.

**Case 3:** We consider a seed solution $u=e^{i\theta }$ with $\theta =ax+bt$, $b=\frac{1+a}{a}\alpha \beta ^2+2\alpha \beta +a\alpha$. We can yield the following systems from Eq. (5)

62 $$\begin{aligned} {\left\{ \begin{array}{ll} -i\lambda ^2\varphi _1-i\lambda e^{i\theta }\varphi _2=\varphi _{1x},\\ -i\lambda e^{-i\theta }\varphi _1+i\lambda ^2\varphi _2=\varphi _{2x}, \end{array}\right. } \end{aligned}$$

without loss of generality, we assume that $\sigma =-1$, $\varphi _1=m e^{c_1 x}$, $\varphi _2=ne^{c_1 x - i\theta }$, then Eq. (62) is solved by

63 $$\begin{aligned} {\left\{ \begin{array}{ll} n=\frac{1\pm \sqrt{s}-2\lambda ^2}{2\lambda }m,\\ c_1=\frac{1\pm \sqrt{s}}{2i}. \end{array}\right. } \end{aligned}$$

We can obtain $s=1+4\lambda ^4$. We derive the system through Eq. (5),

64 $$\begin{aligned} {\left\{ \begin{array}{ll} \left( \frac{i\alpha \beta ^2}{2}-i\eta ^2\right) \psi _1+\left( \frac{i\alpha \beta ^2}{2\lambda }-i\alpha \lambda \right) e^{i\theta }\psi _2=\psi _{1t},\\ \left( \frac{i\alpha \beta ^2}{2}-i\alpha \lambda \right) e^{-i\theta }\psi _1+\left( i\eta ^2-\frac{i\alpha \beta ^2}{2}\right) \psi _2=\psi _{2t}, \end{array}\right. } \end{aligned}$$

without loss of generality, we assume that $\psi _1=p e^{s_1 t}$, $\psi _2=q e^{s_1t - i\theta }$, $\alpha =1$, $\beta =-1$, $\eta =\sqrt{\alpha }(\lambda -\frac{\beta }{2\lambda })$, then Eq. (64) is solved by

65 $$\begin{aligned} {\left\{ \begin{array}{ll} p=\frac{i-2i\lambda ^2}{4i\lambda ^4+2i\lambda ^2+4\lambda ^2s_1+i}q,\\ s_1=\frac{-(40i\lambda ^4+8i\lambda ^2)\pm \sqrt{z^2-64\lambda ^4y}}{32\lambda ^4}, \end{array}\right. } \end{aligned}$$

we can obtain *z* and *y* as following : $z=40i\lambda ^4+8i\lambda ^2$, $y=16\lambda ^8-24\lambda ^6-8\lambda ^5-8\lambda ^4-4\lambda ^3-8\lambda ^2+1$.

It is easy to find two basic solutions for Eqs. (63) and (65):

66 $$\begin{aligned} \left( \begin{array}{c} \phi \\ \psi \end{array}\right) =\left( \begin{array}{c} C_5e^{\frac{(\lambda +\lambda \sqrt{Q})x+(3-4\sqrt{9\lambda ^2-\Delta _2})t}{2i\lambda }} \\ C_6e^{\frac{2\lambda (1+\sqrt{Q})x+(3-4\sqrt{9\lambda ^2-\Delta _2})t}{2i\lambda }-2i\theta } \end{array}\right) , \end{aligned}$$

we can obtain that : $\Delta _2=\lambda ^2(\alpha ^2\beta ^4-4\alpha \eta ^2\beta ^2+6\alpha \beta ^2+4\eta ^4-12\eta ^2-4\alpha ^2\beta ^2+4\alpha ^2\lambda ^2) +\alpha \beta ^4,$ $C_5=\frac{-2\alpha \lambda ^2+\alpha \beta ^2}{\alpha \lambda \beta ^2},$ $C_6=\frac{2\lambda ^2-\sqrt{Q}-1}{2\lambda },$ $Q=3-4\sqrt{9\lambda ^2-\Delta _2}$.

According to Eq. (10), we obtain

67 $$\begin{aligned} M_j=e^{\frac{(\lambda +\lambda \sqrt{Q})x}{2i\lambda }+F_j-2i\theta },~~~1\le j \le 2N, \end{aligned}$$

with $$\nu _j^{(i)}=e^{F_j}$$ $$(1\le i\le 2, 1\le j \le 2N)$$.

(I) We take $N = 1$ with $\lambda =\lambda _j$ (j = 1, 2) and derive the 1-breather solutions of the GNLS equation (4) as following:
  68 $$\begin{aligned} \widetilde{u}(x,t)=e^{i\theta }+\frac{\Delta B_{1}}{\Delta },\quad \widetilde{r}(x,t)=-\widetilde{u}^*(x,t), \end{aligned}$$
  with
  69 $$\begin{aligned} \Delta =\left| \begin{array}{cc} \lambda _1^2&{}M_1\lambda _1\\ \lambda _2^2&{}M_2\lambda _2 \end{array}\right| ,\, \Delta B_1=\left| \begin{array}{cc} \lambda _1^2&{}-1\\ \lambda _2^2&{}-1\end{array}\right| ,~\Delta C_1= \left| \begin{array}{cc} -M_1&{}\lambda _1^2M_1\\ -M_2&{}\lambda _2^2M_2 \end{array}\right| . \end{aligned}$$
(II) We take $N = 2$ in the *N*-times DT with $\lambda = \lambda _j (j=1, 2, 3, 4)$. The linear algebraic system (9) leads to the 2-breather solutions of GNLS Eq. (4) as following:
  70 $$\begin{aligned} \widetilde{u}(x,t)=e^{i\theta }+\frac{\Delta B_{2}}{\Delta },~~~ \widetilde{r}(x,t)=-\widetilde{u}^*(x,t), \end{aligned}$$
  with
  71 $$\begin{aligned} \Delta =\left| \begin{array}{cccc} \lambda _1^2&{}\lambda _1^4&{}M_1\lambda _1&{}M_1\lambda _1^3\\ \lambda _2^2&{}\lambda _2^4&{}M_2\lambda _2&{}M_2\lambda _2^3\\ \lambda _3^2&{}\lambda _3^4&{}M_3\lambda _3&{}M_3\lambda _3^3\\ \lambda _4^2&{}\lambda _4^4&{}M_4\lambda _4&{}M_4\lambda _4^3 \end{array}\right| , \Delta B_2=\left| \begin{array}{cccc} \lambda _1^2&{}\lambda _1^4&{}M_1\lambda _1&{}-1\\ \lambda _2^2&{}\lambda _2^4&{}M_2\lambda _2&{}-1\\ \lambda _3^2&{}\lambda _3^4&{}M_3\lambda _3&{}-1\\ \lambda _4^2&{}\lambda _4^4&{}M_4\lambda _4&{}-1\end{array}\right| , \Delta C_2=\left| \begin{array}{cccc} \lambda _1&{}-M_1&{}\lambda _1^2M_1&{}\lambda _1^4M_1\\ \lambda _2&{}-M_2&{}\lambda _2^2M_2&{}\lambda _2^4M_2\\ \lambda _3&{}-M_3&{}\lambda _3^2M_3&{}\lambda _3^4M_3\\ \lambda _4&{}-M_4&{}\lambda _4^2M_4&{}\lambda _4^4M_4 \end{array}\right| . \end{aligned}$$

**Figure 4:**
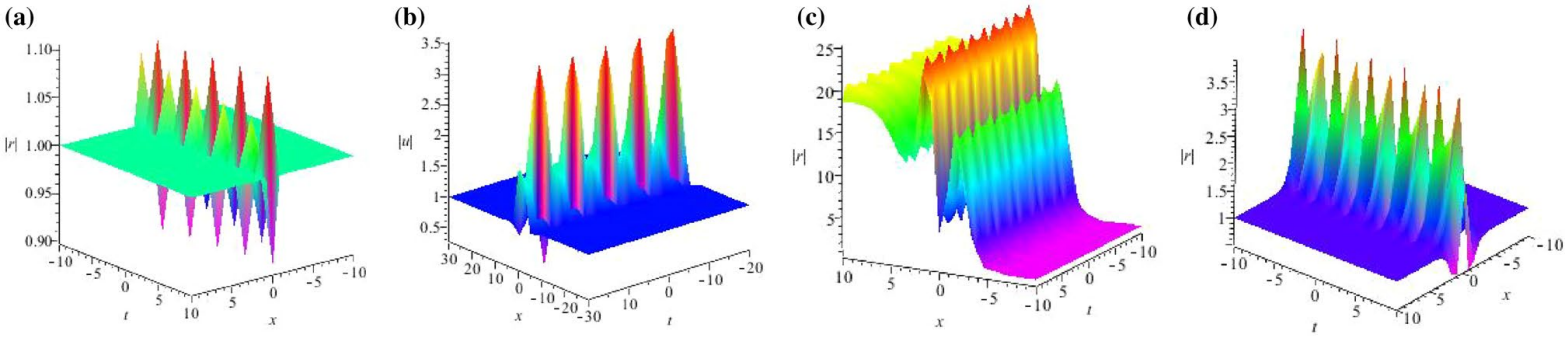
Profiles of intensity distribution (**a**) $|\widetilde{r}(x,t)|$ of Eq. (68) with parameters $\lambda _1=-0.3+5i,\lambda _2=0.3+4i,\alpha =1,\beta =-1,a=-1,b=3,\sigma =-1,F_1=i,F_2=2i$; (**b**) $|\widetilde{u}(x,t)|$ of Eq. (68) with parameters $\lambda _1=0.5i,\lambda _2=0.3i,\alpha =1,\beta =-1,a=-1,b=3,\sigma =-1,F_1=i,F_2=2i$; (**c**) $|\widetilde{r}(x,t)|$ of Eq. (70) with parameters $\lambda _1=0.5i,\lambda _2=-0.3i,\lambda _3=0.2i,\lambda _4=-0.4i,\alpha =1,\beta =-1,a=-1,b=3,\sigma =-1,F_1=i,F_2=2i,F_3=3i,F_4=2i$; (**d**) $|\widetilde{r}(x,t)|$ of Eq. (70) with parameters $\lambda _1=0.03+0.5i,\lambda _2=0.03-0.5i,\lambda _3=0.02+0.3i,\lambda _4=0.02-0.3i,\alpha =1,\beta =-1,a=-1,b=3,\sigma =-1,F_1=i,F_2=2i,F_3=3i,F_4=2i$.

Some breather solutions for GNLS equation (4) with seed $u=\frac{\omega _0}{\beta \gamma _0}e^{-i(\gamma _0^2x+\delta _0t)}$ are shown, we consider $N=2$ and plot their structure figures in Fig. 4.

## Rational wave solutions for GNLS Eq. (4)

In this section, we construct the rational wave solutions of the GNLS Eq. (4). In fact, the rational wave solutions can be obtained by the limits of the eigenfunctions or the limits of the breather solutions.

Based on Eq. (66), we can get a new eigenfunction of the Lax pair (5)

72 $$\begin{aligned} R_1(\varepsilon ) = ( f_1, g_1)^ T , \end{aligned}$$

with

$$\begin{aligned} f_1= & {} C_5e^{\frac{(\lambda +\lambda \sqrt{Q})x+(3-4\sqrt{9\lambda ^2-\Delta _2})t}{2i\lambda }},\,\, g_1=C_6e^{\frac{2\lambda (1+\sqrt{Q})x+(3-4\sqrt{9\lambda ^2-\Delta _2)t}}{2i\lambda }-2i\theta },\\ C_5= & {} \frac{-2\alpha \lambda ^2+\alpha \beta ^2}{\alpha \lambda \beta ^2},\,\, C_6=\frac{2\lambda ^2-\sqrt{Q}-1}{2\lambda },\,\, Q=3-4\sqrt{9\lambda ^2-\Delta _2},\\ \Delta _2= & {} \lambda ^2(\alpha ^2\beta ^4-4\alpha \eta ^2\beta ^2+6\alpha \beta ^2+4\eta ^4-12\eta ^2-4\alpha ^2\beta ^2+4\alpha ^2\lambda ^2) +\alpha \beta ^4, \end{aligned}$$

where $\varepsilon$ is a small parameter, if we fix $\lambda _1 = \frac{1}{2} +\frac{ 1}{2}i$, and let $\lambda = \frac{1}{2} + \frac{1}{2} i + \varepsilon ^2$, then $R_1(\varepsilon )$ can be expanded at $\varepsilon = 1$, so we have

73 $$\begin{aligned} R_1(\varepsilon )=R_1^{[0]}+R_1^{[1]}\varepsilon ^2+R_1^{[2]}\varepsilon ^4+R_1^{[3]}\varepsilon ^6+\cdots \end{aligned}$$

where

74 $$\begin{aligned} R_1^{[0]}=\left( \begin{array}{c} C_5 e^{\frac{Fx+Qt}{i-1}}\\ C_6e^\frac{2Fx+Qt+2\theta (i-1)}{i-1} \end{array}\right) , \end{aligned}$$

and

75 $$\begin{aligned} R_1^{[1]}=\left( \begin{array}{c} \frac{-2i\varepsilon ^2(Fx+Qt)}{(i-1)^2}C_5e^{\frac{Fx+Qt}{i-1}}\\ \frac{4\theta (i-1)-2i[2Fx+Qt+2\theta (i-1)]}{i-1}C_6e^\frac{2Fx+Qt+2\theta (i-1)}{i-1}\end{array}\right) , \end{aligned}$$

with

$$\begin{aligned} Q=3-4\sqrt{9\lambda ^2-\Delta _2},~~F=\lambda +\lambda \sqrt{Q}. \end{aligned}$$

We present the rational wave solution of the GNLS Eq. (4) as following:

76 $$\begin{aligned} u_R=u+\frac{f_1^{[1]}g_1^{[1]^*}(\lambda ^2-\lambda ^{*^2})}{|\lambda |^2(|f_1^{[1]}|^2\lambda +|g_1^{[1]}|^2\lambda ^*)}, \end{aligned}$$

with

$$\begin{aligned} f_1^{[1]}=C_5\frac{-2i\varepsilon ^2(Fx+Qt)}{(i-1)^2},~~~g_1^{[1]}=C_6\frac{4\theta (i-1)-2i[2Fx+Qt+2\theta (i-1)]}{i-1}. \end{aligned}$$

**Figure 5:**
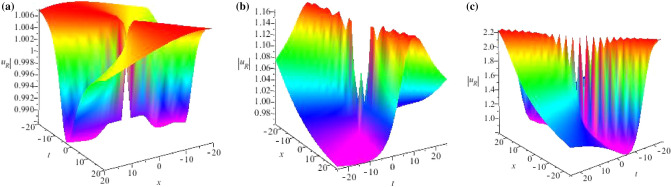
Profiles of intensity distribution (**a**) $|\widetilde{u}_R(x,t)|$ of Eq. (76) with parameters $\lambda =\frac{5}{2}+\frac{1}{2}i,\alpha =-0.3,\beta =0.5,a=-1,b=3$; (**b**) $|\widetilde{u}_R(x,t)|$ of Eq. (76) with parameters $\lambda =\frac{3}{2}+\frac{1}{2}i,\alpha =0.9,\beta =-0.8,a=-1,b=3$; (**c**) $|\widetilde{u}_R(x,t)|$ of Eq. (76) with parameters $\lambda =\frac{1}{2}-i,\alpha =0.6,\beta =-0.6,a=-1,b=3$.

Some rational wave solutions for GNLS equation (4) are shown with the limits of the breather solutions, we plot their structure figures in Fig. 5.

## Data Availability

All data generated or analysed during this study are included in this published article.
